# Skin characterization of diabetes mellitus revealed by polarization-sensitive optical coherence tomography imaging

**DOI:** 10.1117/1.JBO.29.3.036003

**Published:** 2024-03-13

**Authors:** Wei Feng, Lisi Wang, Chun-Jie Liu, Chao Zhang

**Affiliations:** a Central People’s Hospital of Zhanjiang, Zhanjiang Institute of Clinical Medicine, Zhanjiang, China; b Zhanjiang Central Hospital, Guangdong Medical University, Zhanjiang, China; c Huazhong University of Science and Technology, College of Life Science and Technology, Center for Artificial Intelligence Biology, Hubei Bioinformatics & Molecular Imaging Key Laboratory, Key Laboratory of Molecular Biophysics of the Ministry of Education, Wuhan, China

**Keywords:** optical coherence tomography, diabetes, polarization, skin

## Abstract

**Significance:**

Diabetes can lead to the glycation of proteins and dysfunction of skin collagen. Skin lesions are a prevalent clinical symptom of diabetes mellitus (DM). Early diagnosis and assessing the efficacy of treatment for DM are crucial for patient health management. However, performing a non-invasive skin assessment in the early stages of DM is challenging.

**Aim:**

By using the polarization-sensitive optical coherent tomography (PS-OCT) imaging technique, it is possible to noninvasively assess the skin changes caused by diabetes.

**Approach:**

The PS-OCT was used to monitor the polarization characteristics of mouse skin at different stages of diabetes.

**Results:**

Based on a multi-layered adhesive tape model, we found that the polarization characteristics (retardation, optic axis, and polarization uniformity) were sensitive to the microstructure changes in the samples. Through this method, we observed significant changes in the polarization states of the skin as diabetes progressed. This was in line with the detected microstructure changes in skin collagen fibers using scanning electron microscopy.

**Conclusions:**

This study presents a highly useful approach for non-invasive skin assessment of diabetes.

## 1 Introduction

Diabetes mellitus (DM) is a chronic and systemic disease characterized by hyperglycemia.^1^ The number of people with diabetes is increasing annually and is projected to reach 643 million by 2030 and 783 million by 2045.^2^ Several proteins become glycosylated in a high-glycemic environment, which can lead to abnormal cytokine production and various tissue and organ complications.^3^ With a death rate of over 1 million per year, DM is one of the top ten deadliest diseases. The International Diabetic Federation states that one of the most pressing issues in contemporary health care is the early DM diagnosis and monitoring the effectiveness of DM treatment.

Approximately one-third of individuals with diabetes develop skin lesions during the disease, which is one of the most common clinical symptoms of diabetes. Skin abnormalities induced by diabetes typically appear before abnormalities in other internal organs.^4^ Our previous studies have shown that the skin can be used as a target for early preclinical diagnosis of diabetes.^5^^,^^6^ It has been reported that DM leads to a reduction in the lipid and enzyme content of the stratum corneum, as well as the moisture content of the epidermis.^7^ In the dermis, there is an alteration in metalloproteinase expression, repression of collagen expression, and degradation of collagen fibrillar proteins. Furthermore, the balance of proteolytic activity in the dermal extracellular matrix is also disrupted. Based on the traditional pathology section, Tahrani et al.^8^ evaluated the skin thickness, fiber spacing, histochemistry, and fiber dissociation in healthy individuals, diabetics with peripheral neuropathy, and those with diabetic foot ulcers. However, compared to *in vivo* detection, *ex vivo* pathological sections are invasive and do not allow for the dynamic monitoring of skin pathological changes in the same individual. The clinical non-invasive skin assessment for early-stage DM still needs to be developed.

Diabetes causes changes in the composition and structure of the skin, which is closely related to changes in the optical characteristics of the skin. This presents a new opportunity for the development of optical techniques to quantitatively assess skin alterations. It is widely recognized that collagen, a major component of the skin, determines its optical scattering and birefringent properties.^9^ The density and other mechanical properties of the collagen fibers are linked to scattering and birefringence.^10^^–^^12^ Utilizing optical diagnostic techniques is a powerful strategy to enhance the accuracy of diagnosis in modern medicine and advanced medical and biological research. Previous studies have shown that polarization of scattered polarized light changes because of alterations in skin collagen structure,^13^ showing that light polarization is sensitive to structural changes in the skin. In particular, polarization techniques have been used to assess the microstructural alterations in biological tissues caused by conditions such as cancer,^14^ diabetic wounds,^15^ burns,^16^ and others.^17^ Polarization-sensitive optical coherence tomography (PS-OCT) is a functional OCT imaging technique that combines polarization imaging with OCT.^18^^–^^20^ PS-OCT can not only get the traditional OCT tomography images, but also capture the polarization states of the backscattered light from tissue.^19^ It has potential to be a promising noninvasive 3D imaging tool for assessing collagen organization *in vivo*.^21^ PS-OCT imaging will be a valuable tool for the early diagnosis of diabetes.

Here, we used PS-OCT to image a multi-layered tape model to simulate the polarization changes caused by structural alterations. We introduced PS-OCT imaging to monitor the polarization properties of the skin and assess collagen alterations. To characterize dynamic changes in the polarization of diabetic skin, we performed long-term imaging of the dorsal skin at different stages of diabetes. Furthermore, in order to validate the accuracy of PS-OCT assessment of skin alterations, scanning electron microscopy (SEM) was used to investigate the changes in skin collagen fibers. This work presents an important approach for the early clinical diagnosis of DM and for monitoring the effectiveness of diabetic treatment.

## 2 Materials and methods

### 2.1 Type 1 Diabetes (T1D) Model

Adult male Balb/c mice (8 weeks old, 23±2  g) were intraperitoneally administered with streptozotocin (STZ, V900890, Sigma, St. Louis, MO) at a dosage of 200  mg/kg at 4 h after fasting. To allocate sodium citrate buffer, 2.1 g citric acid monohydrate (C1909, Sigma, St. Louis, MO) and 2.94 g sodium citrate (S4641, Sigma, St. Louis, MO) were dissolved in 100 ml distilled water, respectively, and then mixed in proportions of 1:1.32, whose PH was 4.2 to 4.5. Each time before injection, the STZ was dissolved in sodium citrate buffer at 1% (w/v). Intraperitoneal injections should be finished within 15 min after drug preparation. The mice were injected for five consecutive days. After multiple STZ injection, the blood glucose was measured using a glucometer (580, Yuyue Medical Equipment & Supply Co., Ltd., China). Mice with fed blood glucose ≥16.7  mM (300  mg/L) were considered diabetic and selected for imaging. The protocol refers to relevant literature.^22^^,^^23^ Additionally, the mice were divided into five groups (Normal; DM-1 week; DM-2 weeks; DM-3 week; DM-4 week). There were five mice in each group for PSOCT and SEM imaging. For Masson’s trichrome staining experiments, five mice were used in each group. All experimental procedures were carried out in accordance with the Guangdong Provincial Experimental Animal Management Ordinance and the Guangdong Medical University’s guidelines, which had been approved by the Central People’s Hospital of Zhanjiang’s Institutional Animal Ethics Committee.

### 2.2 PS-OCT System

**Fig. 1:**
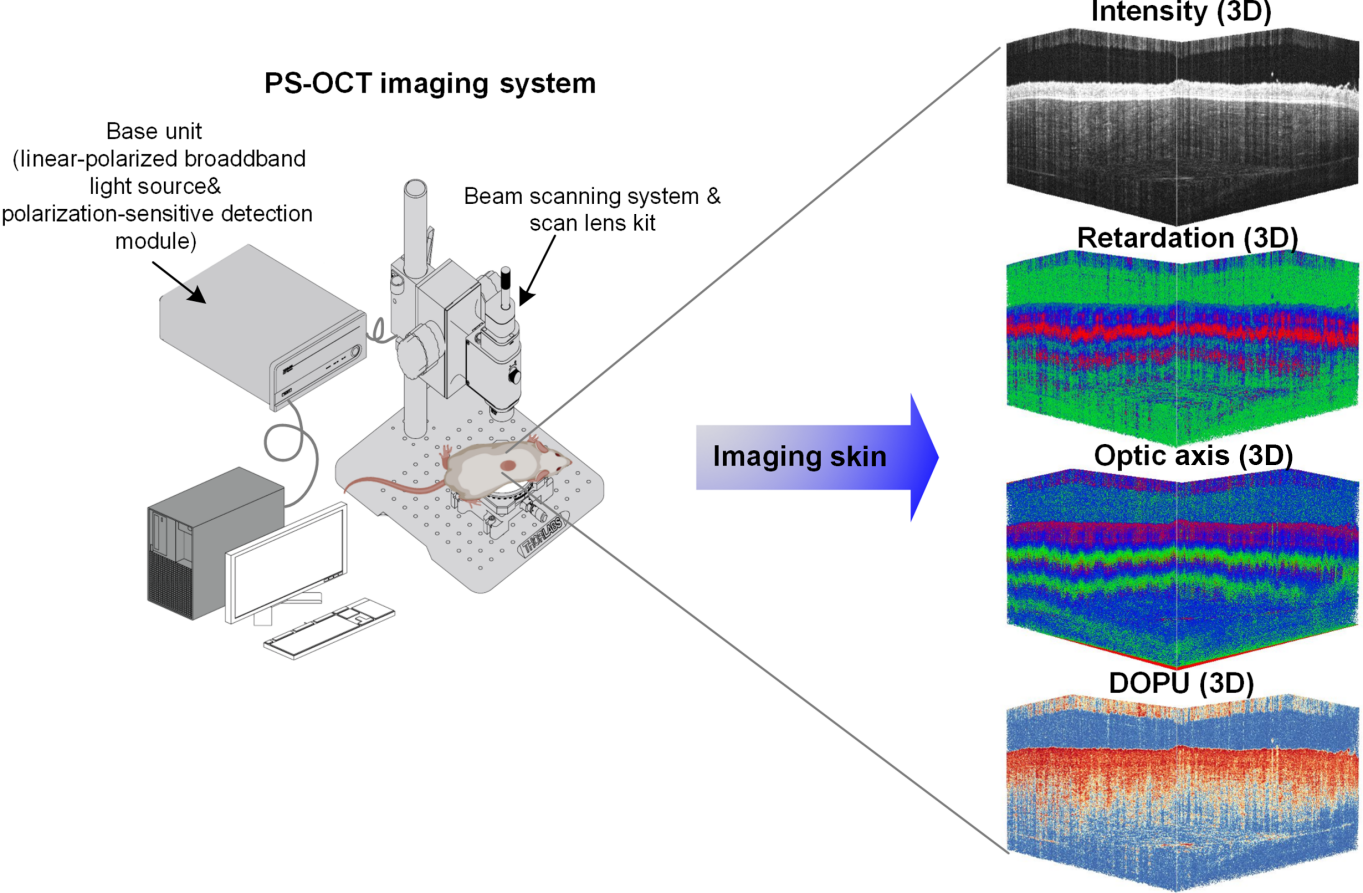
Schematic of PS-OCT imaging system.

We used a commercial PS-OCT imaging system (TEL221PS, Thorlabs Inc., Germany), with a scanning lens (OCT-LK3, 5×), to monitor how the skin structure changes as DM progresses. The central wavelength is 1300 nm, with a bandwidth of 170 nm. The imaging depth and axial resolution in air/water are 3.5  mm/2.6  mm and 5.5  mm/4.2  mm, respectively. This PS-OCT utilizes an incident beam with known polarization and a dual-detector design to incorporate polarization information in 2D cross-sectional and 3D volumetric images of a sample. It controls the polarization incident on the sample and reflects it in the reference arm of the interferometer by using two carefully aligned wave plates. The preserved polarization information is then measured using two detectors. The schematic of this PS-OCT system is shown in Fig. 1.

### 2.3 Evaluation of Polarization Parameters

We utilized the 3D polarization-sensitive mode in the PS-OCT system to capture polarization images and collected intensity and polarization signals from two sensors. The Stokes vectors (I, Q, U, and V) can be determined from the data provided by the two orthogonal polarization states of the interference light for each point. The retardation, optic axis, and degree of polarization uniformity (DOPU) are three advanced polarization characteristics that can be calculated using the Stokes vector to describe the polarization state of light. The formula below can be used to evaluate the polarization parameters

$$\text{Retardation}=\tan^{-1}(\sqrt{I_{0}/I_{1}})\in [0,\pi /2],$$ (1)

$$\text{Optic Axis}=\frac{1}{2}a\;\tan\;2(U,Q)\in [-\pi /2,\pi /2],$$ (2)

$$\mathrm{DOPU}=\sqrt{{\left\langle Q\right\rangle }^{2}+{\left\langle U\right\rangle }^{2}+{\left\langle V\right\rangle }^{2}}\in [0,1].$$ (3)

The intensity of detector-0 and detector-1 is represented by $I_{0/1}$. Q stands for the percentage of either horizontally (Q=−1) or vertically (Q=+1) polarized light. U is proportion of light linearly polarized at 45 deg (U=−1) or linearly polarized at 135 deg (U=+1). V is proportion of left-circularly (V=−1) or right-circularly (V=+1) polarized light. The algorithm specifics are described previously.^24^ ThorImageOCT (version 5.4) is utilized for acquiring and processing polarization images.

Additionally, intrinsic birefringence mainly occurs in regularly ordered fibrillar tissues^25^ The local birefringence (Δn) can be determined from the cumulative phase retardation. It is calculated according to the following equation:^26^

$$\Delta n=\frac{\lambda }{2\pi }\frac{d\delta_{\mathrm{cum}}(z)}{dz}.$$ (4)

The λ is center wavelength of the light used. The $d\delta_{\mathrm{cum}}(z)/dz$ is the local slope of cumulative phase retardation as a function over z position.

### 2.4 SEM for Obtaining the Microstructure of Skin with Development of T1D

For the normal and T1D mice at different stages, $1\;{\mathrm{cm}}^{2}$ skin samples were collected after using a hair removal cream (3221469, Veet, Reckitt Benckiser Group Plc (RB), France), and PBS (>4 times) carefully washed the residual blood. Then, these samples were fixed with 2.5% glutaraldehyde (G5882, Sigma, St. Louis, MO) overnight at 4°C. The samples were rinsed twice in PBS and then fixed for an hour in 1% osmium acid that had been pre-cooled to 4°C. The samples were dehydrated, rinsed twice with PBS, and then treated with dry heat. Finally, the samples were processed by conduction, and a scanning electron microscope (SU8100, Hitachi, Ltd., Japan) was used to capture images of the skin’s microstructure. To determine the structure of collagen fibers in the skin dermis, it was necessary to scan the cross-section of the skin sample at a magnification of 500. According to the cross-sectional image, the microscopic structure of the collagen fibers was scanned. About ∼250  μm below the epidermis (magnification = 15k/40k). Additionally, we also evaluated the orientation of the skin collagen fibers using the ImageJ/FIJI directionality plugin.

### 2.5 Masson’s Trichrome Staining

The collagen in the skin of normal mice was observed through pathological sections at different time points after the application of hair removal cream. The dorsal skin tissue was cut into ∼0.5  cm×0.5  cm pieces and then promptly washed with cool saline to remove blood. The samples were then fixed in 4% neutral paraformaldehyde, dehydrated, and embedded in paraffin. After cutting into 4-μm sections, they were stained with Masson’s trichrome.

## 3 Results

### 3.1 Effect of Structural Modifications on Polarization State in Multi-Layered Model

**Fig. 2:**
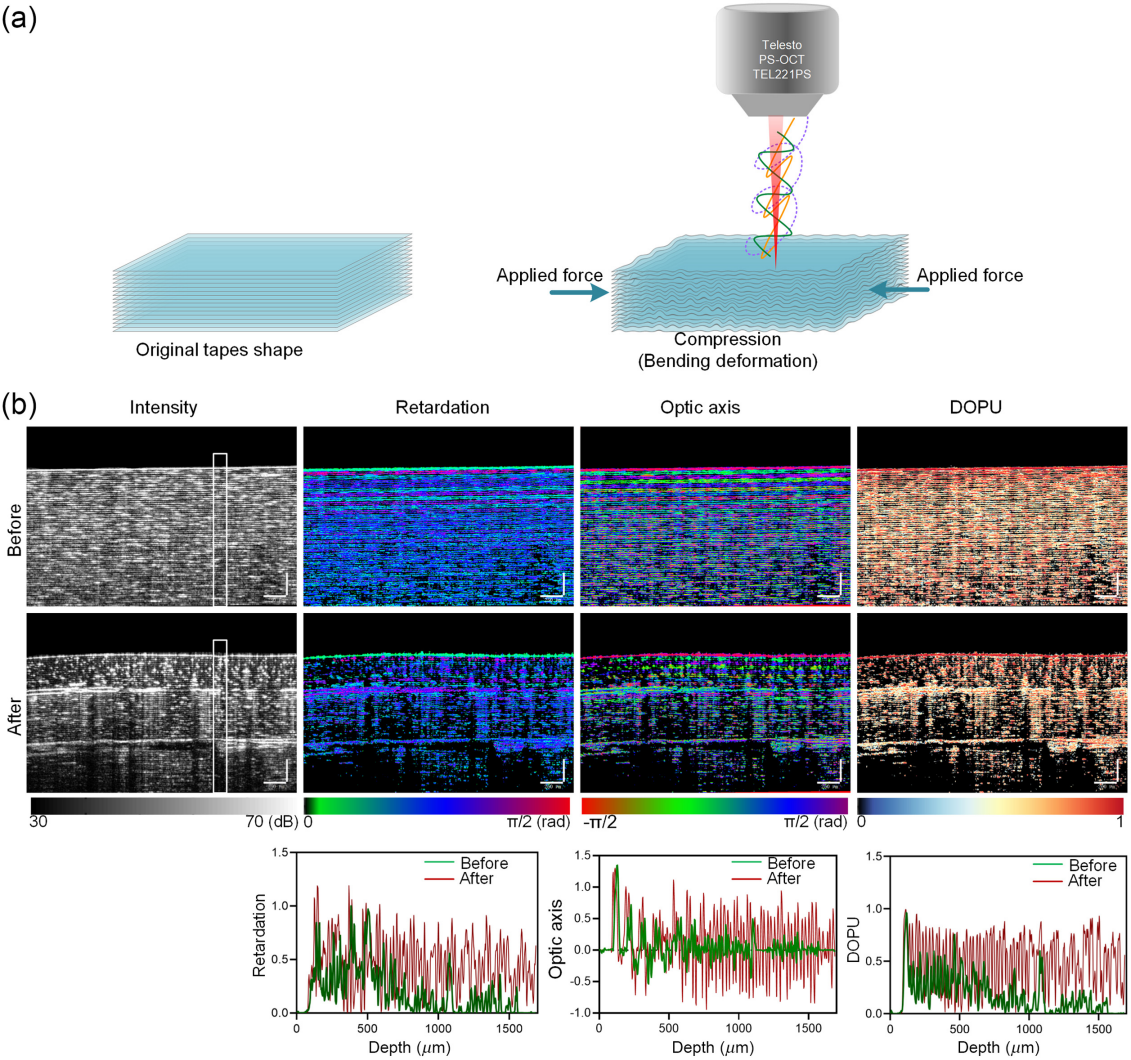
PS-OCT imaging of the multi-layered tape model. (a) Schematic diagram of the multilayer tape model before and after deformation. (b) The intensity, retardation, optic axis, and DOPU images of multi-layered adhesive tape model before/after compression (scale bar is 250  μm). The corresponding profiles of the polarization parameters (retardation, optic axis, DOPU, and local birefringence) of the white rectangle in intensity images along the depth direction. The dashed line was the fitted curve for the corresponding data, and the average residual is shown in Table S1 in the Supplementary Material.

The birefringence of standard adhesive tapes was evaluated in a recent study.^27^ Here, a multi-layered tape made of polyethylene and adhesive (each layer ∼0.05  mm thick) was used to simulate layered structure of biological tissues. The laminated structure will deform under compression, and we used this multi-layered phantom to simulate the impact of structural modifications on polarization [Fig. 2(a)]. The polarization images were captured before and after deformation. Figure 2(b) shows the typical cross-sectional images. The average residual (the distance from the data to the fitted curve) was calculated to assess the fluctuation amplitude of the corresponding polarization parameters (Table S1 in the Supplementary Material). In the undeformed phantom, there was a consistent change in retardation, optic axis and DOPU. The deformed phantom exhibited significant changes in retardation, optic axis and DOPU. Additionally, we also assessed the local birefringence. The profiles of changes in retardation, optic axis, and local birefringence at different depths indicated that the residual decreased following the deformation. It indicated that the fluctuation amplitude decreased. The Stokes parameters in pixel-neighborhood were homogeneous before deformation, and the phantom refractive index was also uniform, as the DOPU was reasonably close to 1. The profiles of DOPU change at various depths also demonstrated that the magnitude of fluctuation decreased after the deformation. The compression-induced deformation could lead to changes in the original compact and ordered layer structure of the tape, and there were even gaps between some layers. The disorganized distribution of layers and presence of gaps affected light propagation, leading to changes in polarization characteristics. These results suggest that PS-OCT imaging can effectively assess changes in structure of layered tissues.

### 3.2 Changes of Skin Polarization State with Development of DM

**Fig. 3:**
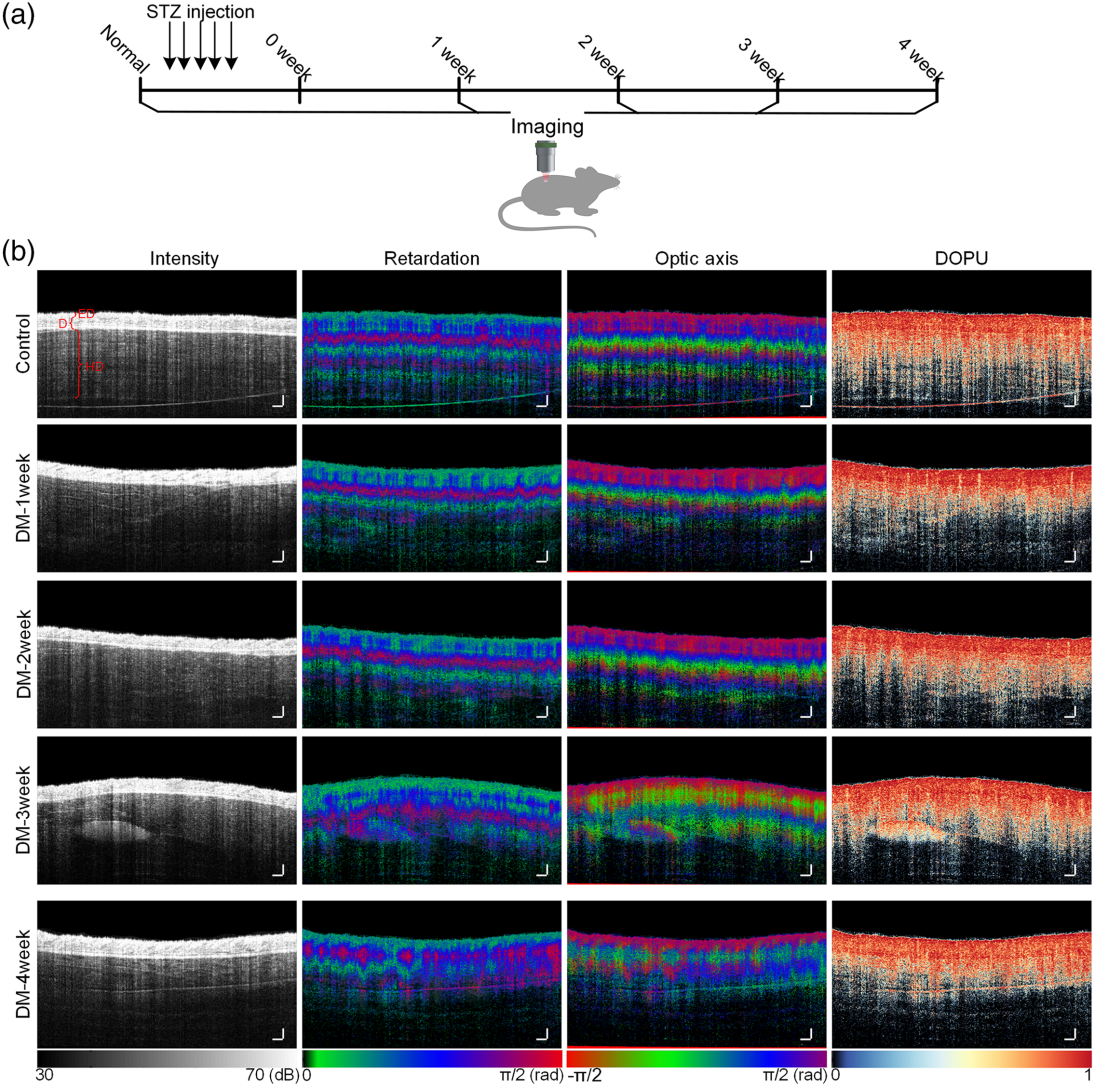
Monitoring the dorsal skin of DM mice by PS-OCT. (a) Graphic illustration of the experiment workflow. (b) The intensity, retardation, optic axis, and DOPU images of skin at different stages of DM. (E, epidermis; D, dermis; HD, hypodermis; scale bar is 250  μm.)

Here, we established the STZ-induced T1D model and used PS-OCT to monitor polarization images of the mouse dorsal skin at different stages of DM [Fig. 3(a)]. Figure 3(b) shows the sectional views of intensity, retardation, optic axis and DOPU. The OCT intensity images revealed the structure of skin, including the epidermis, dermis, and hypodermis, with areas of low intensity that likely represent vessels or fat. For the normal mouse, the regular “layer-like structure” was observed in the retardation and optic axis images of skin. The DOPU of epidermis and dermis was higher than that of the hypodermis. According to polarization images, the skin’s polarization characteristics did not change significantly after the diabetic mice were kept in a state of hyperglycemia for 2 weeks. However, for mice in DM-3 week and DM-4 weeks, there were changes in skin retardation and optic axis images, and the “like-layered structures” observed in images of normal skin became disordered.

**Fig. 4:**
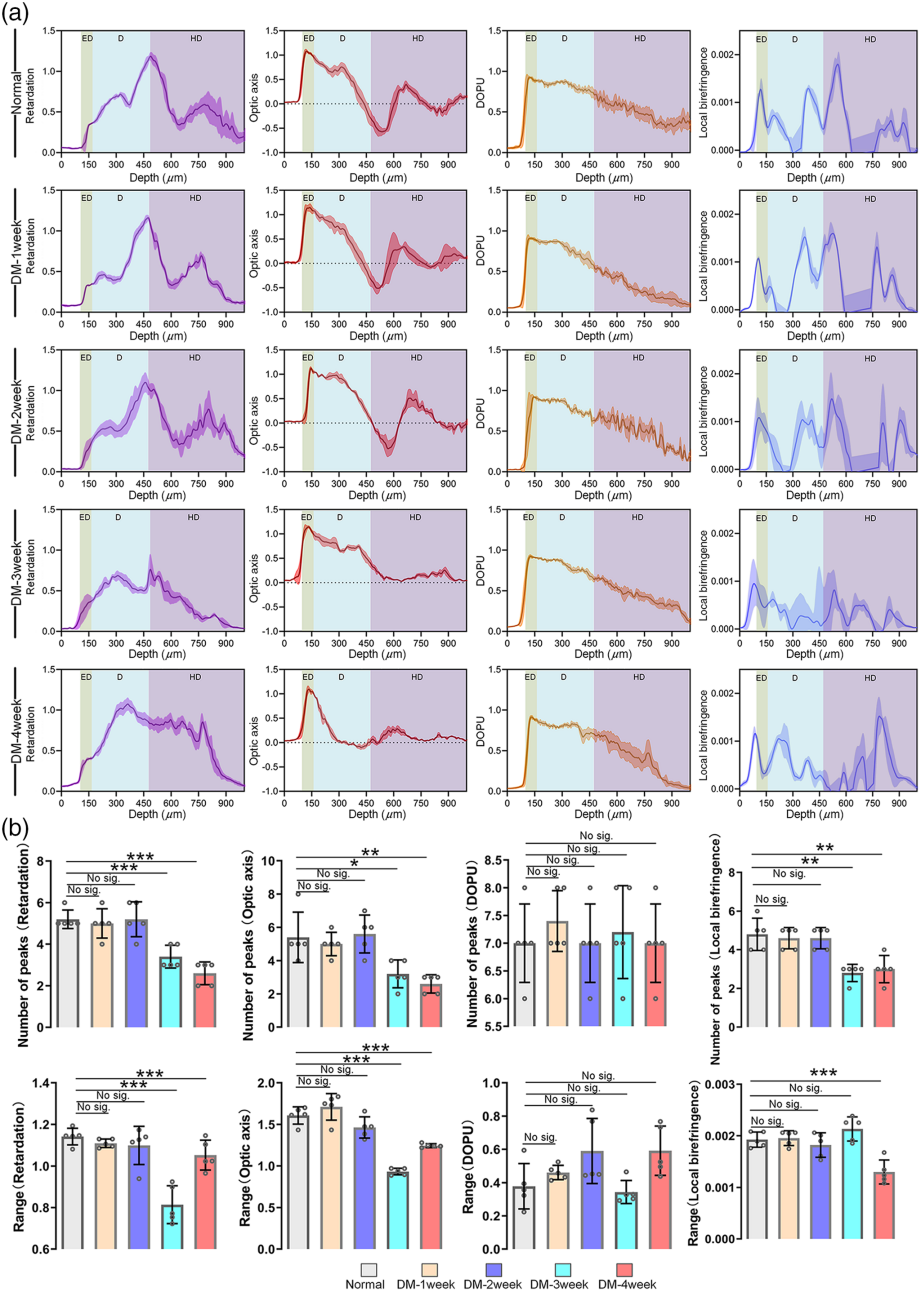
Analysis of DM-induced changes in polarization characteristic parameters (retardation, optic axis, DOPU, and local birefringence) based on the PS-OCT images. (a) The curve and the width of shadow are mean and standard deviation, respectively (E, epidermis; D, dermis; HD, hypodermis; n=5, mean ± standard deviation.) (b) The number of peaks and range (max-min) of the curve in (a) (Depth: 100 to 500  μm; n=5, error bars represent the standard deviation, one-way ANOVA analysis and two-tail t-test. Not significant: p>0.05; *p<0.05; **p<0.01; ***p<0.001).

Furthermore, based on polarization images collected by PS-OCT, we analyzed profiles of skin polarization characteristics along the depth, as shown in Fig. 4(a). We also assessed the local birefringence based on cumulative phase retardation with the depth. Moreover, the number of peaks and range of values were used to further quantify changes in polarization parameter curves within the skin depth range (depth: 100 to 500  μm) [Fig. 4(b)]. For normal mice, there were multiple peaks in the retardation and optical axis curve, which was similar to the DM-1 week and DM-2 week groups. For DM-3 week and DM-4 week, the retardation and optical axis curves changed significantly, with decreases in range values and the number of peaks. It implied that the amplitude of their fluctuations changed. Additionally, compared with the normal group, there was also a significant decrease in the number of peaks of local birefringence curves in the DM-3 week and DM-4 week groups. The reduction in the fluctuations of the curve might be linked to the changes in skin structure induced by diabetes. This was consistent with the model test results above regarding effects of altered layer structure on polarization parameters. However, when compared with the normal mice, there was no significant change in DOPU curve for diabetic mice. These results suggest that non-invasive PS-OCT imaging can be used to evaluate skin structure disorders caused by diabetes.

### 3.3 Assessment of DM Induced Changes in Skin Collagen

**Fig. 5:**
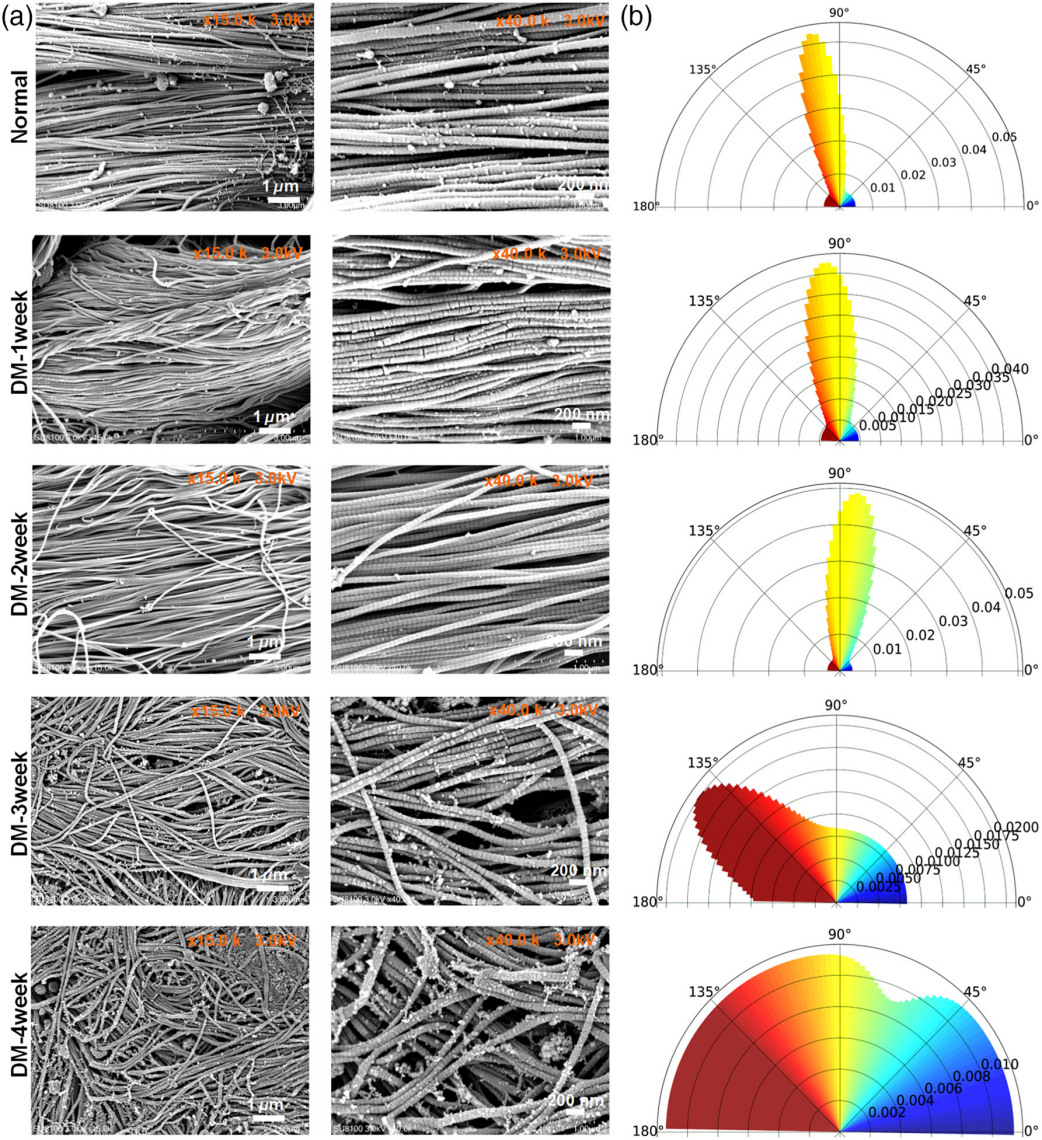
The changes in skin collagen structure at different DM stages. (a) SEM images of skin. (b) Typical quantitative assessment of the collagen orientation from 0 deg to 180 deg. (The polar axis represents the proportion of collagen in each direction.)

The rich collagen fibers are essential for maintaining the structure and function of skin. The alterations in morphology and diversity of collagen fiber orientation can be effectively revealed through birefringence. Figure 4(a) and Table S2 in the Supplementary Material suggested that, in comparison with normal mice, the local birefringence values showed no significant changes in DM-1 week and DM-2 week mice at the imaging depth of 300 to 500  μm. However, these values decreased significantly for DM-3 week and DM-4 week mice. Accordingly, the results above suggest that changes in collagen fiber structure could be the cause of alterations in skin polarization images. Further, SEM was used to assess the microscopic structure of skin at various stages of DM, as illustrated in Fig. 5(a). To assess the disorder of collagen, we also utilized directionality analysis to evaluate the percentage of collagen fibers oriented 0 deg to 180 deg as shown in Fig. 5(b). It was observed that collagen fibers in the normal, DM-1 week and DM-2 week mice exhibited an orderly alignment, arranged neatly in a relatively consistent direction. However, for DM-3 week and DM-4 week, the distribution of collagen fibers gradually became disorganized, orienting in all directions. These results were consistent with the variations in local birefringence. Furthermore, to determine whether hair removal cream would affect skin’s collagen structure, we conducted Masson’s trichrome staining of the normal skin at various time points following application of the hair removal cream (Fig. S1 in the Supplementary Material). The results indicated that a basic hair removal treatment did not have a significant impact on skin structure. Therefore, it indeed indicates that changes in polarization characteristics could effectively reflect diabetes-induced alterations of skin structure.

## 4 Discussion

Skin, as the body’s largest organ,^28^ is the first line of defense against invasion. Diabetes can damage the structure and function of skin^29^ and lead to complications in many organs and tissues, including retinopathy, neuropathy, kidney disease, cardiovascular disease, peripheral vascular disease, cerebrovascular disease, and diabetic foot.^30^ Nowadays, skin changes are recognized as common clinical signs of diabetes and often appear before disease becomes evident. It may serve as a potential starting point for an accurate diagnosis. Furthermore, skin changes in development of diabetes could be associated with issues in various internal organs.^4^ Thus, skin imaging and analysis are crucial for the clinical management of diabetes.

Presently, the primary focus of DM-induced skin disorders lies in the structural composition and microvasculature of skin.^6^^,^^31^ Diabetes-induced glycation end products cross-linked with skin collagen can be stimulated by a 370 nm laser to emit 440 nm fluorescence. Analyzing variations in skin autofluorescence signals is expected to assist in the diagnosis of diabetes.^32^ Therefore, detecting the skin autofluorescence signal is an important aspect of evaluating the impact of diabetes on the skin.^33^^–^^40^ However, it cannot be used to detect the glycosylation levels of non-fluorescent molecules. The excitation spectra of glycation end products overlap with those of other fluorescent molecules, such as tryptophan.^41^ Furthermore, the process of skin glycosylation is age-dependent, which introduces some limitations as an assessment method. Therefore, there is still a need to develop precise, non-invasive imaging methods to assess diabetes-induced changes in skin structure and composition.

Polarization-sensitive hyperspectral imaging has been used to monitor the structural and physiological changes of human skin abnormalities.^42^ Recently, Dremin et al.^43^ developed a novel polarization-enhanced neural network-assisted hyperspectral imaging method to assess blood content, blood oxygenation, and structural changes in the skin of patients with DM. They found significant changes in both skin polarization parameters and blood content in individuals with diabetes. It was consistent with our findings regarding the changes in skin polarization parameters caused by diabetes, as well as our previous report that DM could result in skin microcirculation dysfunction.^5^ Therefore, a comprehensive analysis of skin vascular function and structural parameters is more conducive to evaluating the progression of diabetes. Furthermore, several studies have shown that diabetes can lead to significant changes in skin collagen.^44^^,^^45^ Argyropoulos et al.^46^ utilized atomic force microscopy to observe the nanoscale morphology of collagen fibrils in the skin. The results indicated that DM could cause collagen fibers to fragment and become disorganized, which aligns with our findings (Fig. 5). In this study, we utilized PS-OCT to assess skin microstructural changes caused by diabetes. PS-OCT offers 3D optical tomography capabilities, in contrast to polarization-sensitive hyperspectral imaging, making it a powerful tool for investigating 3D structural characteristics of tissues. Tissue birefringence properties are related to the retardation and optic axis contrasts,^47^ which are sensitive to the collagen structure. Thus, many studies of skin PS-OCT imaging have primarily focused on retardation, optic axis, and DOPU.^47^^–^^49^

The attenuation of light by various layers of biological tissue may affect the measurement results of polarization.^50^ The intensity profile along depth in OCT intensity images of the tape shows fluctuations caused by its multilayered structure, and the signal intensity does not exhibit a noticeable overall decay [Fig. S2(a) in the Supplementary Material]. Moreover, the OCT intensity signal in the dermal layer of the skin does not show noticeable attenuation either, while a clear signal intensity decay is observed for the hypodermis at depths below ∼500  μm [Fig. S2(b) in the Supplementary Material]. This means that the optical attenuation coefficient is almost constant within the dermal layer. Therefore, our quantitative analysis about the variation curves of the polarization parameters is conducted within the depth range of ∼100 to 500  μm in the skin. Here, we found that DM could lead to changes in retardation and the optic axis, and the DOPU might not be very sensitive to changes in skin structure during the early stages of diabetes. The study demonstrated the feasibility and usefulness of PS-OCT as a non-invasive imaging technique for identifying changes in skin structural characteristics. As far as we know, we are the first to demonstrate the feasibility of using PS-OCT to assess skin structure disorders in an animal model of diabetes. We will attempt to validate it with individuals who have diabetes in the future.

## 5 Conclusion

We introduced PS-OCT to assess skin dysfunction caused by DM. PS-OCT imaging of a multilayer structure model showed that polarization characteristics were sensitive to changes in tissue microstructure. Furthermore, it was used to monitor the skin of diabetic mice, and there was a noticeable change in polarization maps with the onset of diabetes. Similarly, the results of SEM revealed that the organization of collagen fibers in the skin became disordered with the onset of diabetes, which was in line with the PS-OCT imaging. This study will provide a potential approach for non-invasive clinical skin assessment of DM.

## Supplementary Material



## Data Availability

Full images generated in this paper can be obtained from the authors upon request.
