# Supplementary material for: Skin characterization of diabetes mellitus revealed by polarization-sensitive optical coherence tomography imaging
Source: J Biomed Opt. 2024 Mar 13;29(3):036003. doi: 10.1117/1.JBO.29.3.036003 (PMC10933388; doi:10.1117/1.JBO.29.3.036003)
Supplement: Supplementary file 1 [file JBO_029_036003_SD001.pdf]

# Supplementary Materials

## Skin characterization of diabetes mellitus revealed by polarization-sensitive optical coherence tomography imaging

**Wei Feng,[a,b] Lisi Wang, [a,b] Chun-jie Liu[c] and Chao Zhang [a,b,*]**

[a] Zhanjiang Institute of Clinical Medicine, Central People's Hospital of Zhanjiang, Zhanjiang, Guangdong 524045, China.

[b] Zhanjiang Central Hospital, Guangdong Medical University, Zhanjiang, Guangdong 524045, China.

[c] Center for Artificial Intelligence Biology, Hubei Bioinformatics & Molecular Imaging Key Laboratory, Key Laboratory of Molecular Biophysics of the Ministry of Education, College of Life Science and Technology, Huazhong University of Science and Technology, Wuhan, 430074, China

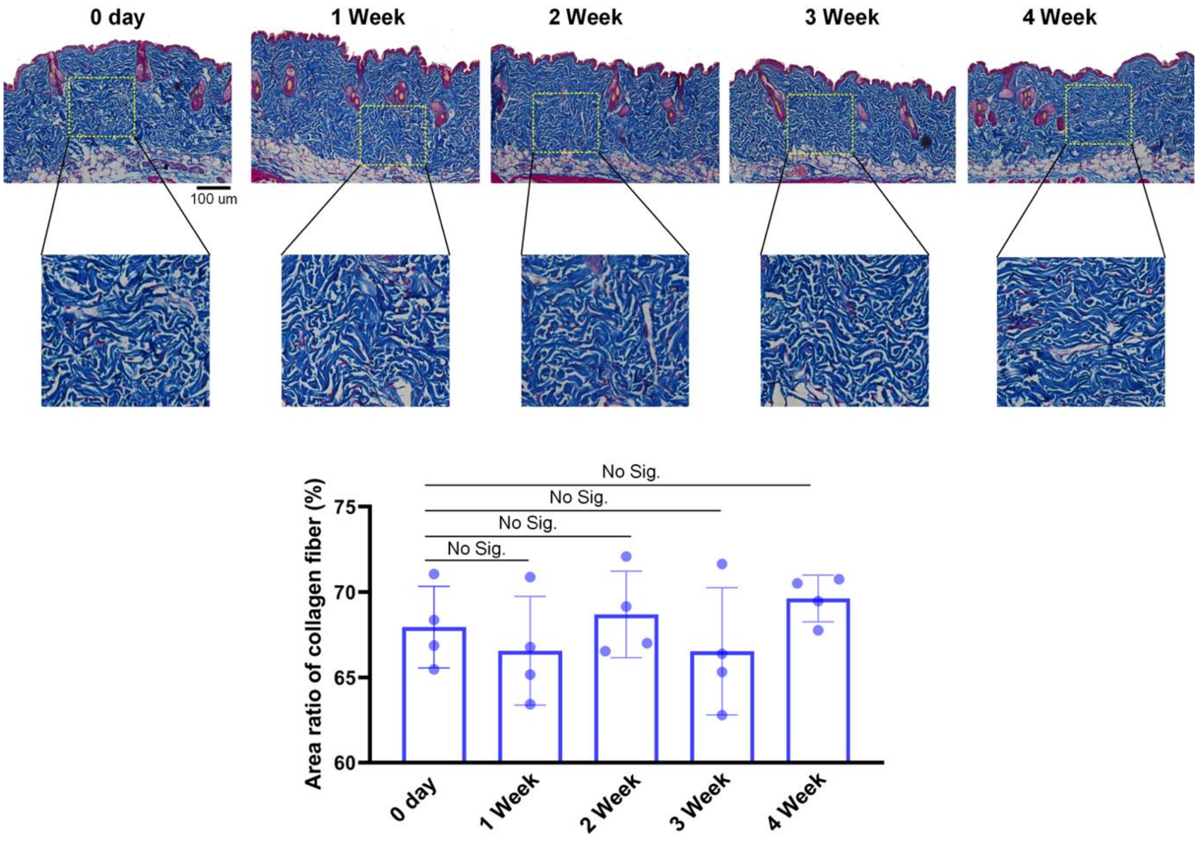


Fig S1. Masson staining of normal mice skin after hair removal using the hair removal cream. The area ratio of collage was analyzed. (n=4, Error bars represent the standard deviation, One-way ANOVA analysis and two-tail t-test, No Sig.: $p > 0.05$)

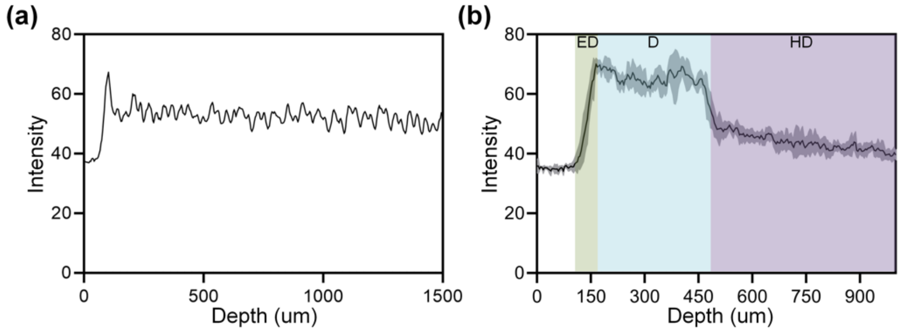


Fig S2. The intensity profile along depth in OCT intensity images of the tape (a) and the skin (b) (The curve and the width of shadow are mean and standard deviation, respectively. E: Epidermis, D: Dermis, HD: Hypodermis) (n=5, mean ± standard deviation).

Table S1. The residual (distance from the data to the fitted curve of Fig. 2 (b)). (mean ± standard error))

| | **Retardation** | **Optic Axis** | **DOPU** | **Local Birefringence** |
|---|---|---|---|---|
| **Before** | 0.097±0.0039 | 0.197±0.0042 | 0.112±0.0037 | $0.000263 \pm 1.25\times10^{-5}$ |
| **After** | 0.069±0.0035 (***) | 0.091±0.0071 (***) | 0.071±0.0030 (***) | $0.000226 \pm 9.67\times10^{-6}$ (*) |

One-way ANOVA analysis and two-tail t-test, No Sig.: $p > 0.05$; *: $p < 0.05$; **: $p < 0.01$; ***: $p < 0.001$ (Before deformation v.s. After deformation)

Table S2. The maximum values of local birefringence values at the imaging depth of 300µm -500µm. (n=5, mean ± standard deviation))

| | **Normal** | **DM-1 week** | **DM-2 week** | **DM-3 week** | **DM-4 week** |
|---|---|---|---|---|---|
| **Maximum value** | $0.0013 \pm 6.99\times10^{-5}$ | $0.0017 \pm 1.78\times10^{-4}$ (*) | $0.0015 \pm 2.55\times10^{-4}$ (No Sig.) | $9.7303\times10^{-4} \pm 3.36\times10^{-4}$ (*) | $6.6209\times10^{-4} \pm 5.18\times10^{-5}$ (***) |

One-way ANOVA analysis and two-tail t-test, No Sig.: $p > 0.05$; *: $p < 0.05$; **: $p < 0.01$; ***: $p < 0.001$ (DM groups v.s. Normal)
